# Conceptions of Learning and Teaching for Faculty Who Teach Basic Science

**DOI:** 10.1007/s40670-021-01264-4

**Published:** 2021-03-15

**Authors:** Helena Carvalho, Francis C. Dane, Shari A. Whicker

**Affiliations:** 1 Department of Basic Science Education, Virginia Tech Carilion School of Medicine, Roanoke, VA USA; 2 Department of Psychology, Radford University, Roanoke, VA USA; 3 Office of Continuing Professional Development, Carilion Clinic and Virginia Tech Carilion School of Medicine, Roanoke, VA USA

**Keywords:** Teaching approaches, Active learning, Active teaching, Teaching methodology

## Abstract

**Introduction:**

Conceptions of learning and teaching refer to what faculty think about teaching effectiveness. Approaches to teaching refer to the methods they use to teach. Both conceptions and approaches range from student-centered/learning-focused (active learner engagement) to teaching-centered/content-focused (passive learner engagement). This study explored how faculty teaching experience influenced faculty conceptions and their approaches to teaching. The authors hypothesized that more experienced educators appreciate and apply active learning approaches.

**Methods:**

The authors used a cross-sectional survey to collect anonymous data from the Basic Science faculty at Virginia Tech Carilion School of Medicine (VTCSOM). The survey included the Conceptions of Learning and Teaching scale (COLT; Jacobs et al. 2012) and demographic information. They assessed instrument reliability with Cronbach’s alpha and examined relationships between variables with correlation and chi-square and group differences with ANOVA.

**Results:**

Thirty-eight percent (50/130) of faculty responded to the survey. COLT scores for student-centered (4.06 ± 0.41) were significantly higher (*p* < 0.001) than teacher-centered (3.12 ± 0.6). Teacher-centered scores were lower (*p* < 0.05) for younger (30–39, 2.65 ± 0.48) than older faculty (50–59, 3.57 ± 0.71) and were negatively correlated with using multiple teaching methods (*p* = 0.022). However, 83% (39/50) reported using both traditional lectures and active approaches.

**Discussion:**

Faculty conceptions about teaching showed appreciation for active learning, but a tendency to use traditional teaching methods interspersed with student-centered ones. Teaching experience was not related to faculty conceptions but was related to their teaching approaches. The amount of time dedicated to teaching was related to the appreciation of active learning, and young teachers were more student-oriented.

**Supplementary Information:**

The online version contains supplementary material available at 10.1007/s40670-021-01264-4.

## Introduction

Ideas about the purpose of teaching can be divided into two broad categories: content-focused and learning-focused [1, 6]. A content-focused, faculty-centered approach focuses on the content and relies on the faculty to transmit knowledge to the students who are, in essence, passive recipients of the transmitted information. On the other hand, in the learning-focused, student-centered approach, the intended purpose is to improve the students’ learning process and considers the students responsible for their own learning. Faculty approaches to teaching can also be viewed as a continuum ranging from faculty-centered to student-centered.

When faculty focus purely on transmitting content knowledge, students are more likely to report superficial learning. Conversely, when faculty adopt student-centered approaches, students have reported significantly deeper learning [1]. The ways by which faculty approach teaching has been shown to affect students’ learning and academic performance. When presented with opportunities to engage in active discussion in a traditional physiology laboratory, for example, students who engaged in the active learning process improved knowledge acquisition and reported increased satisfaction [2].

While it is known that faculty concepts of learning and teaching influence their approaches to teaching, what faculty think and say about teaching is not always reflected in their teaching practice. In one study, for example, Laksov et al. [3] reported that, despite 25% of faculty members’ endorsing a constructivist teaching philosophy, only 12.5% reported applying constructivist teaching principles in the classroom. Translating the way faculty think about teaching into practice has been described as a challenge regardless of their concepts of learning and teaching [4].

It is important to know what the faculty conceptions of learning and teaching are and whether they are consonant or dissonant with their approach to teaching [5]. A consonant profile is one in which conceptions of learning and teaching are consistent with teaching practices, either faculty-centered or student-centered. A dissonant profile consists of incompatible combinations of conceptions and practices, in which both content-focused and learning-focused conceptions and approaches are combined in one way or another. When a faculty member uses a consonant profile, students’ learning outcomes have been shown to be of higher quality than when a faculty member employs a dissonant profile [6]. Faculty members who do not know that their teaching methods are dissonant with their concepts of learning and teaching may be less effective in the classroom, so it is important to find out whether methods and concepts match among the faculty. This may be particularly important for Basic Science faculty because they are among the first to interact with medical students in the classroom, setting the stage for the remainder of the students’ medical school experience. Also, there is some evidence [4, 7] that a faculty member’s experience influences conceptions of learning and teaching. Given the impact of faculty member’s teaching on a student’s involvement in learning, it is important to assess the relationship between faculty concepts of learning and teaching and their choice of teaching methods.

This study aimed to examine the Basic Science faculty member’s conception of learning and teaching (what they believe about teaching) and their approaches to teaching (how they teach) at the Virginia Tech Carilion School of Medicine (VTCSOM). We hypothesized that faculty members with more experience would use more student-centered approaches to teaching with a variety of teaching methods and would score lower on teacher-centeredness.

## Methods

The Virginia Tech IRB approved this minimal-risk study. We collected data during August–September 2018 from 130 faculty who teach Basic Science for 1st- and 2nd-year students at VTCSOM. VTCSOM’s hybrid curriculum is designed to engage students with a student-centered approach that includes problem-based learning, lectures, workshops, and anatomy laboratory classes.

Faculty members were invited via e-mail to respond to an anonymous, online survey via REDCap®. The survey included demographic information (gender, age, academic rank, and degree), items about teaching practices, and experience and time dedicated to teaching (appendix). The survey also included the validated Conceptions of Learning and Teaching scale (COLT) instrument [7]. The COLT is an 18-item scale containing three independent subscales: teacher-centeredness (TC), which assesses how important the respondent perceives their role as a teacher (*α* = 0.73); appreciation of active learning (AL), which assesses how the respondent values students’ discussing, elaborating, and interpreting learning material (*α* = 0.57); and orientation to professional practice (OP), which assesses the respondent’s valuation of integrating future professional practice into teaching (*α* = 0.63). All COLT responses were rated on a five-point response continuum ranging from 1 (strongly disagree) to 5 (strongly agree). The survey takes about 5 min to complete, and faculty had three opportunities to respond as reminders were sent twice to individuals who did not reply. Consent was obtained by survey completion.

We examine internal consistency with Cronbach’s alpha to assess reliability for our sample. We examined relationships between variables with regression, correlation, and chi-square. We employed analysis of variance (ANOVA) to examine group differences for normally distributed variables with effect size estimated by partial eta-squared, η_p_^2^; the Kruskal-Wallis ANOVA test for skewed variables; the Jonckheere-Terpstra test for skewed variables with ordered alternatives; and the Mann-Whitney *U* test for nonparametric, two-group comparisons. We tested normality with Shapiro-Wilk’s test. We completed all analyses with SPSS version 25 (IBM, Armonk, NY). We described all normally distributed variables with mean ± standard deviation (SD) and others with median ± interquartile range.

## Results

### Demographics

Of the 130 faculty members invited to participate, 50 (38%) responded, of whom 23 (46%) were women (3 chose not to disclose). Table 1 shows the respondents’ diversity with respect to academic rank, degree type, years teaching, and percentage of time spent teaching in a year. The reported number of hours per year spent teaching ranged from 1 to 110: median = 6, interquartile range = 18, mean = 17, *SD* = 25.4.

**Table 1:** Frequency and (percentages) of categories for demographic variables

**Academic Rank**	**None** **5 (10)**	**Instructor** **1 (2)**	**Asst. Prof** **24 (48)**	**Assoc. Prof** **12 (24**)	**Professor** **8 (16)**
Age (years)	< 30 1 (2)	30–39 5 (11)	40–49 20 (43)	50–59 8 (17)	60 + 13 (28)
Degree	MSN 1 (2)	PhD 12 (24)	PharmD 4 (8)	DO 1 (2)	MD 31 (63)
Percent of Time Spent Teaching	< 5 1 (2)	5–15 11 (23)	16–30 13 (27)	31–50 12 (25)	> 50 11 (23)
Years Teaching	< 5 5 (10)	5–10 13 (26)	> 10 30 (60)	Unknown 2 (4)	

**Fig. 1:**
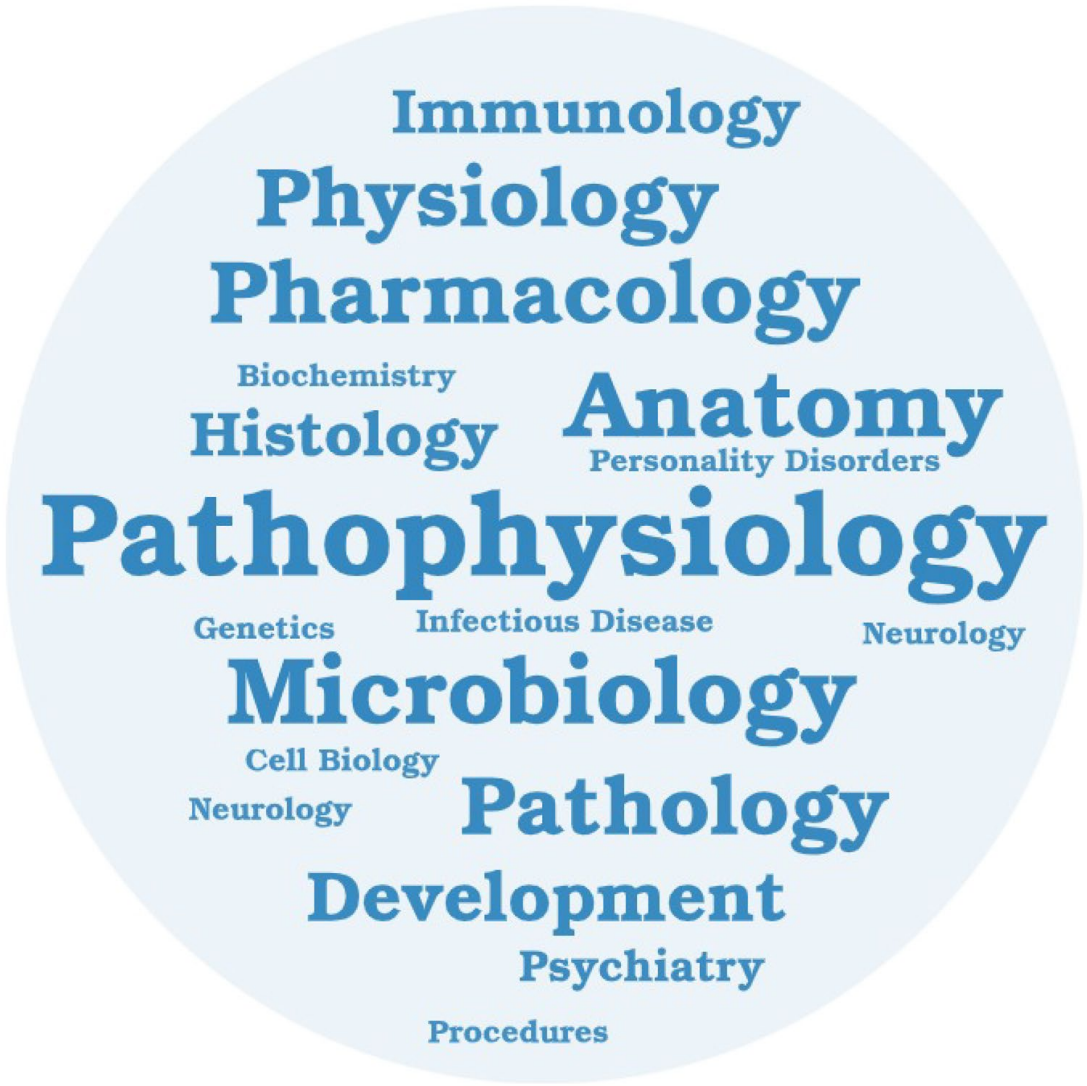
Faculty report of subjects they teach in size relative to the number who reported teaching each subject

Almost half of the respondents with a Ph.D. taught more than 50% of their time in Basic Science, while only 16% of the physicians reported teaching the same amount in Basic Science. The majority of the physicians’ teaching time was in clinical areas. PharmDs taught less than 5% in Basic Science. Those who dedicated a greater percentage of time teaching taught more hours per year (*p* = 0.038), especially those who dedicated more than 50% of their time to teaching. Figure 1 displays a word cloud of the subjects that respondents reported teaching, wherein it is apparent that not all reported subjects are within the Basic Science purview (e.g., procedures, personality disorders, and psychiatry). It is clear that our respondents teach beyond the Basic Sciences topics, which was represented in their responses.

Four respondents did not provide information about their teaching methods, but among those who did, only seven (15%) reported using exclusively traditional (e.g., lecture, laboratory) activities. Lecture was the most popular method (Table 2). Among the demographic variables, only the number of hours spent teaching per year was related to using exclusively traditional teaching practices (*p* = 0.023). Faculty who used exclusively traditional practices (median = 2.0 ± 5.0) spent much less time teaching during the year than those who employed other student-centered teaching methods (median = 7.5 ± 22.1).

**Table 2:** Teaching methods selected from drop-down list. Participants chose all that apply to their teaching modalities

Teaching method	Number (%)
Lecture*	41 (82%)
Case-based learning	32 (64%)
Small-group discussion	13 (26%)
Independent learning	12 (24%)
Large-group discussion	12 (24%)
Laboratory*	10 (20%)
Team-based learning	10 (20%)
Demonstration	9 (18%)
Concept map	5 (10%)
Conference	5 (10%)
Peer teaching	4 (8%)
Game	3 (6%)
Self-directed learning	3 (6%)
Dramatization	2 (4%)
Tutorial	2 (4%)
Simulation	1 (2%)
Reflection	1 (2%)
Quizzes	1 (2%)
Questions and problems	1 (2%)

Faculty reported using more than one modality, so total counts exceed 50 respondents and total percentages exceed 100

*Teacher-centered methods at VTCSOM

### Concepts of Learning and Teaching

Teacher-centeredness (TC) and orientation to professional practice (OP) achieved acceptable reliability (*α* = 0.731, 0.730, respectively), while appreciation of active learning (AL) evidenced lower reliability (*α* = 0.608) than is considered acceptable. TC was negatively correlated with both AL (*r* = −0.31, *p* = 0.03) and OP (*r* = −0.20, *p* = 0.18), while AL and OP were significantly correlated (*r* = 0.60, *p* < 0.001).

**Fig. 2:**
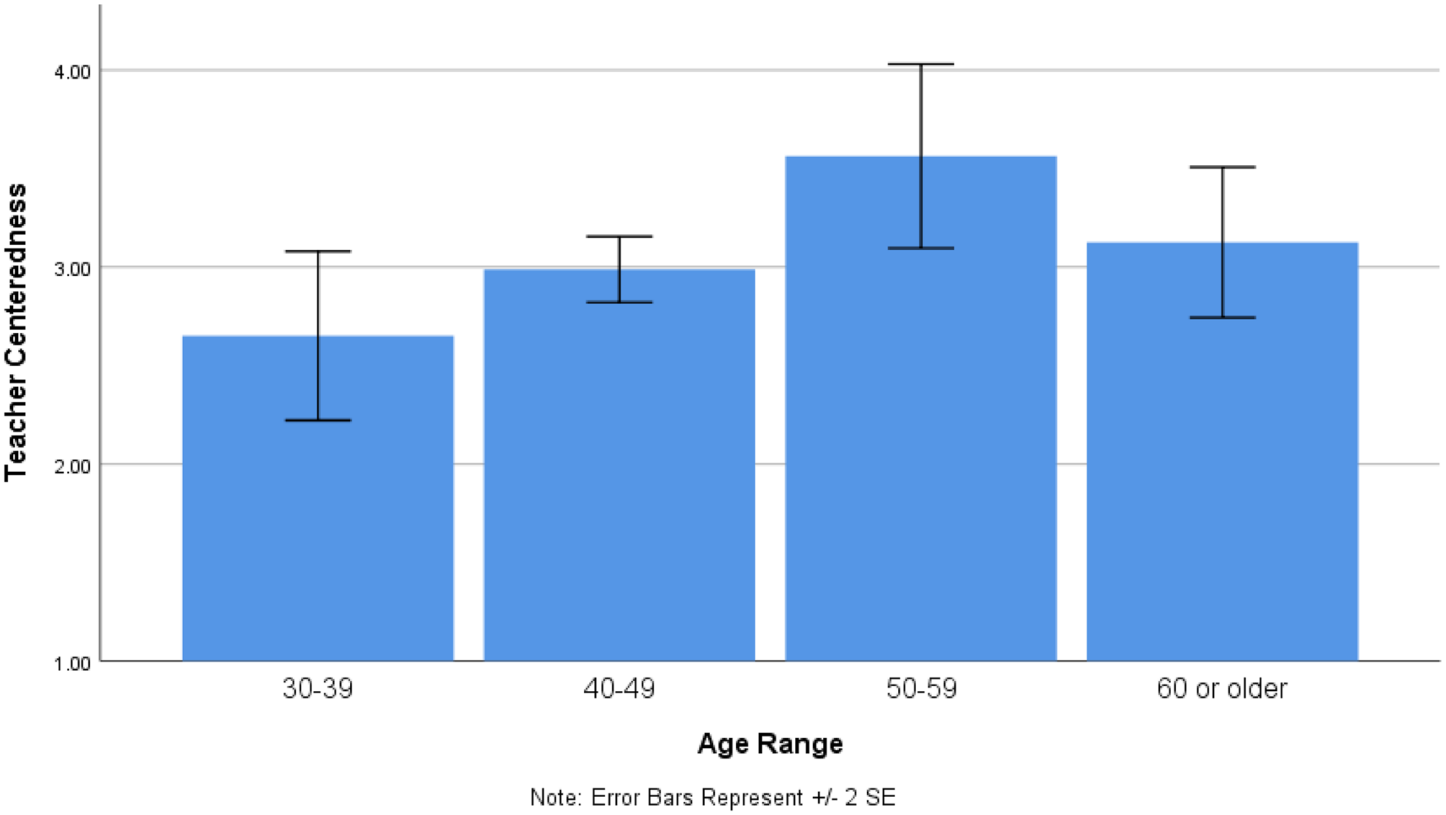
Mean scores on teacher-centeredness subscale as a function of age range

Overall, faculty scored higher on AL (4.06 ± 0.41) and OP (4.2 ± 0.45) than on TC (3.12 ± 0.6) regardless of gender, academic rank, degree, years of teaching, or percentage of time allocated to teaching Basic Science (*p* < 0.001, partial *η*_*p*_^*2*^ = 0.588). In the repeated-measures ANOVA, there were significant differences among the age categories (*p* = 0.037, *η*_*p*_^*2*^ = 0.189), but the age *X* subscale interaction was not significant (*p* = 0.202). Analysis of the separate subscales produced a significant age effect only for TC (*p* = 0.027, *η*_*p*_^*2*^ = 0.20), in which the 50–59 age group evidenced a significantly higher mean score than the other age groups (Fig. 2).

Faculty who scored lower on teacher-centeredness report using a greater variety of teaching methods (*r* = −0.323, *p* = 0.022), but the diversity of teaching methods was not correlated with the other COLT subscale scores (*p*s > 0.5).

**Fig. 3:**
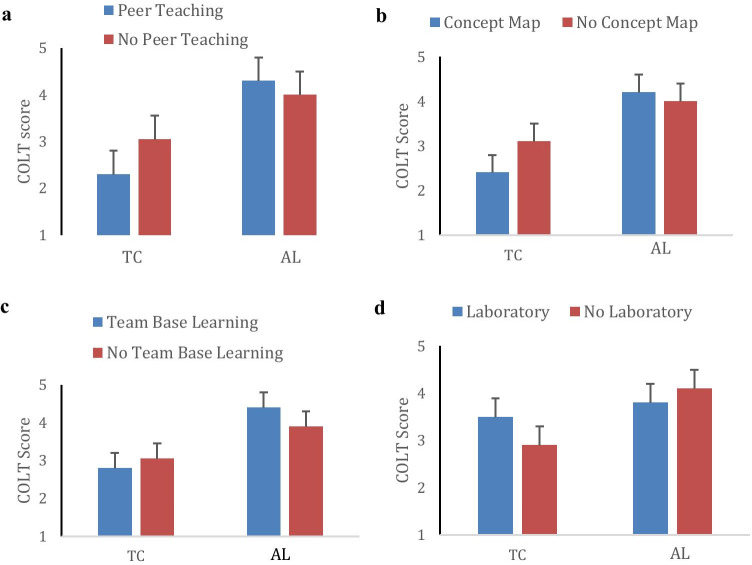
Comparison of COLT subscales for faculty who use one type of teaching modality with faculty who do not use it. **a** Peer teaching. **b** Concept map. **c** Team-based learning. **d** Laboratory. TC teacher-centered subscale, AL appreciation of active learning subscale

We also analyzed the COLT subscales by separately comparing faculty who used only one type of teaching method to those who did not use that specific modality. A pattern emerged that was statistically significant (*p* < 0.05) in four of the teaching methods listed (Fig. 3). Faculty who exclusively used either peer teaching, concept maps, or team-based learning scored significantly lower in teacher-centered (*p* < 0.05) and slightly higher in active-learning subscales than those who did not use these teaching methods. In contrast, faculty who used only laboratory teaching scored significantly higher in teacher-centered (*p* < 0.05) and slightly lower in active learning COLT subscales than those who did not. This analysis was not possible for the other teaching methods as they were not used exclusively.

### Teaching Experience and Choice of Teaching Methods

Faculty members who currently dedicate more time to teaching, regardless of how many years teaching, indicated making less use of lectures (*r* = 0.483; *p* = 0.001). Faculty members who hold a higher academic rank, regardless of their age, also reported using more teaching modalities (*r* = 0.401; *p* = 0.006).

## Discussion

Our data did not support our hypothesis that faculty members with more experience would demonstrate more appreciation for active learning using a variety of teaching methods and scoring lower in teacher-centeredness. We did not find a relationship between years of teaching and subscale scores. Instead, we found that the Basic Science faculty at VTCSOM expressed conceptions of learning and teaching that were more appreciative of active learning and more oriented to professional practice than they were interested in teacher-centeredness. Despite this, the vast majority of the faculty still reported using traditional lectures at least some of the time when they teach, even though the lecture might be interspersed with other student-centered teaching methods. There appears to be some evidence of dissonant [6] teaching profiles among the respondents.

However, the COLT teaching-centered subscale was significantly correlated with faculty use of teaching methods, suggesting that faculty thoughts about teaching (conceptions of learning and teaching) were related to how they teach (teaching methods). Faculty whose concepts of learning and teaching showed more appreciation of active learning than faculty-centered teaching also showed a consonant approach to teaching, as their choice of teaching methods—particularly with respect to the use of concept maps, team-based learning, and peer-teaching—was learning-focused and student-oriented. Also, faculty who revealed a more teacher-centered conception used more traditional teaching approaches such as lectures and laboratories. At VTCSOM, laboratory activity is anatomy dissection, which despite being hands-on is predominantly teacher-centered because a great deal of faculty direction and supervision is necessary to ensure that the cadavers are preserved for the entire term.

The 50–59-year-old faculty members scored highest on teaching-centeredness but the same as other age groups on appreciation for active learning. This suggests this group could be dissonant on their conceptions versus their practice of teaching [5, 6]. One possible explanation could be that these faculty may have learned in a teacher-centered environment but recognize the importance of teaching using student-centered approaches in which a variety of teaching methods are stimulated and expected. An example of this is when a faculty member includes learning-focused activities such as multiple-choice questions in lecture but fails to allow enough time for students’ participation. Another example of dissonance between teaching conceptions and practice is the struggle some experience when they try student-centered or active-learning methods but continue to feel responsible for covering the same amount of content they would deliver in a traditional lecture. Other than the 50–59 age group, however, there is no specific age group who stand out concerning scores on the COLT, nor was there a difference between physicians and other degree holders.

Teaching experience was not correlated with concept of teaching but was related to how faculty members teach and their choice of teaching methods. Instead of years of experience teaching, we found the amount of time dedicated to teaching during the work year was correlated with faculty choice of teaching methods. A greater variety of teaching methods, in turn, was negatively correlated with scores on the teacher-centered subscale. This suggests that simply accumulating years of teaching is not enough to influence what faculty think about teaching. Time dedicated to teaching and exposure to active learning modalities may be important factors, whereas only being exposed to traditional teaching methods such as lectures might perpetuate the use of a teaching-centered mindset. Jacobs et al. (2016), for example, found that lower scores on teacher-centered measures are related to teaching multiple disciplines. As faculty spend more time teaching and teach different disciplines, they may develop the confidence to depart from traditional methods [8] or incorporate student-centered activities into lectures [9].

Alternately, more time dedicated to teaching may expose faculty members to more student feedback that stimulates a more sophisticated way of thinking about teaching. Engaging in a variety of student-centered activities allows the educator to reach a larger number of students with different learning styles. Medical students might suggest varied teaching styles, which have been shown to be preferred [9, 10]. Faculty members who try new teaching methods may find that students become more engaged and improve their performance [2], which could further encourage them to try additional methods.

Limitation of our data includes the limited size of our sample. The return of 38% response rate may be considered low, but it was all we could achieve after three administration attempts. We recognize the concern regarding self-report and lack of information about previous participation in faculty development. Also, we collected data in one medical school where the expectation is for the faculty to be student-centered. We would like to expand our research and compare our data with other institutions with a focus both on student and/or faculty-centered curricula.

We appreciate that there are additional questions that could have been included in the survey, including more information on perception of the institution’s teaching culture, institutional support of specific teaching methods, availability of technology for innovative active teaching, rewards for teaching effectively using specific methods, and the influence of student evaluations of choice of teaching methods. Future directions for our work will be to consider these questions as well as to incorporate student learning outcomes and explore mechanisms for application to faculty development initiatives.

## Conclusion

Faculty conceptions of learning and teaching were related to teaching methods as respondents with a low score on teacher-centeredness used more teaching methods and were younger, while faculty with a higher score on teacher-centeredness used fewer teaching modalities and were older.

Our data suggest that faculty members who dedicate more time to teaching have more student-centered conceptions of learning and teaching as well as approaches to teaching. More important than the number of years teaching, per se, is the number of hours dedicated to teaching in a given period.

## Supplementary Information

Below is the link to the electronic supplementary material.

Supplementary file1 (DOCX 28 KB)
