# Supplementary material for: Conceptions of Learning and Teaching for Faculty Who Teach Basic Science
Source: Med Sci Educ. 2021 Mar 15;31(2):745–51. doi: 10.1007/s40670-021-01264-4 (PMC8368879; doi:10.1007/s40670-021-01264-4)
Supplement: Supplementary file 1 — Supplementary file1 (DOCX 28 KB) [file 40670_2021_1264_MOESM1_ESM.docx]

COLT questionnaire

**Response scale:**  1-strongly disagree; 2-disagree; 3-neutral; 4-agree; 5-strongly agree

**Factor 1: Teacher Centeredness**

1. Students should first master Basic Science knowledge before they can formulate their own learning goals.

2. I think that in small group learning the tutor determines what the students should learn, instead of the students determining their own learning goals.

3. Students learn best when the learning process is guided by an expert who has an overview of the field of interest.

4. When students discuss a topic without a tutor being present, they do not know at the end of the session if the questions have been answered correctly.

5. There is a logical sequence to learning.

6. As a teacher I have to indicate clearly what is important and what is less important for the students to know.

7. I think that as an expert in my field I am eminently suitable to transmit my knowledge to students and that students should not have to look up that knowledge for themselves.

8. When students collaborate, they teach each other the wrong things.

**Factor 2: Appreciation of Active Learning**

9. Students learn a great deal by explaining subject matter to each other.

10. Learning materials and teaching should invite students to come up with examples to illustrate the subject matter.

11. Small group learning motivates students to study.

12. I think it is more important for students to be able to analyze and critically appraise subject matter than to memorize facts.

13. I think it is important that students advise each other about the best ways to study.

**Factor 3: Orientation to Professional Practice**

14. I think it is important that educational assignments are derived as much as possible from the students’ future professional practice.

15. Being introduced to the day-to-day practice of their future profession motivates students to learn.

16. It is a good learning outcome when students demonstrate that they can apply their knowledge during activities in situations in professional practice.

17. I think that interactions between me and my students are an important aspect of my teaching.

18. Discussing topics with each other helps students to learn how to deal with different points of view, so as to gain a deeper understanding.

**Demographic Information**

1. Gender:

a. male

b. female

c. prefer not to disclose

2. Highest degree earned (e.g. PhD; MD):

3. Age:

a. Below 30

b. 30-39

c. 40-49

d. 50-59

e. Older then 60

4. How many years of direct teaching experience do you have?

A: < 1 year

B: 1 - 5 years

C: 5 – 10 years

D: > 10 years

5. What percentage of work time to do you spend teaching in general (all levels of learners)?

A: < 5%

B: 5 -15%

C: 15-30 %

D: 30-50%

E: > 50%

Answer the remaining questions based on your current **Basic Science** teaching (morning sessions) for M1 and M2 at VTCSOM.

6. How many hours of Basic Sciences classes do you teach per year?

7. Which Basic Science subject do you teach? Select all that apply.

a. anatomy

b. physiology

c. histology

d. pathology

e. development

f. pharmacology

g. pathophysiology

h. microbiology

i. immunology

j. other: please list

8. Which type of classroom activities do you use while teaching Basic science? Select all that apply.

A: lectures

B: case-based instruction

C: concept map

D: conference

E: demonstration

F: large group discussion

G: small group discussion

H: games

I: Independent learning

J: Laboratory

L: Peer teaching

M: reflection

N: role play/dramatization

O: simulation

P: self-direct learning

Q: Team Based Learning

R: Tutorial

S: workshop

T: other: please list

9. Academic Rank (e.g. assistant, associate, professor, etc):
